# Current hepatitis E virus seroprevalence in Swiss blood donors and apparent decline from 1997 to 2016

**DOI:** 10.2807/1560-7917.ES.2018.23.35.1700616

**Published:** 2018-08-30

**Authors:** Christoph Niederhauser, Nadja Widmer, Magdalena Hotz, Caroline Tinguely, Stefano Fontana, Gabrielle Allemann, Mauro Borri, Laura Infanti, Amira Sarraj, Jörg Sigle, Michèle Stalder, Jutta Thierbach, Sophie Waldvogel, Tina Wiengand, Max Züger, Peter Gowland

**Affiliations:** 1 Interregional Blood Transfusion SRC, Berne Switzerland; 2 Servizio Trasfusionale CRS della Svizzera Italiana, Lugano, Switzerland; 3 Blood Transfusion Service SRC, Fribourg, Switzerland; 4 Blood Transfusion Service Beider Basel, Basel, Switzerland; 5 Blood Transfusion Service SRC Neuchâtel/Jura, Neuchâtel, Switzerland; 6 Blood Transfusion Service SRC, Aargau/Solothurn, Switzerland; 7 Blood Transfusion Service SRC Nordostschweiz, St. Gallen, Switzerland; 8 Blood Transfusion Service SRC, Geneva, Switzerland; 9 Blood Transfusion Service SRC Zentralschweiz, Luzern, Switzerland; 10 Blood Transfusion Service SRC Thurgau, Münsterlingen, Switzerland

**Keywords:** hepatitis E, virus seroprevalence, blood donors, Switzerland

## Abstract

Hepatitis E virus (HEV) is a virus of emerging importance to transfusion medicine. Studies from several European countries, including Switzerland, have reported high seroprevalence of hepatitis E as a consequence of endemic infections. Published HEV seroprevalence estimates within developed countries vary considerably; primarily due to improved diagnostic assays. The purpose of this study was to investigate the seroprevalence of anti-HEV IgG in Swiss blood donations. **Methods:** We used the highly sensitive Wantai HEV IgG EIA and assessed regional distribution patterns. We analysed age- and sex-matched archive plasma dating back 20 years from canton Bern to investigate recent changes in HEV seroprevalence levels. **Results:** On average, 20.4% (95% confidence intervals: 19.1–21.8) of the 3,609 blood samples collected in 2014–16 were anti-HEV IgG positive; however, distinct differences between geographical regions were observed (range: 12.8–33.6%). Seroprevalence increased with age with 30.7% of males and 34.3% of women being positive donors over > 60 years old. Differences between sexes may be attributed to dissimilarities in the average age of this group. Within the specified region of the Bern canton, overall prevalence has declined over two decades from 30.3% in 1997/98 to 27.0% in 2006 and 22.3% in 2015/6. **Conclusions:** HEV seroprevalence in Switzerland is high, but has declined over the last decades. The result shows that primarily endemic HEV infections occur and that current blood products may pose a risk to vulnerable transfusion recipients. Nucleic acid screening of all blood products for HEV will begin in November 2018.

## Introduction

Hepatitis E virus (HEV), is a small, non-enveloped virus belonging to the family *Hepevirus,* was first described in 1978 during a non-A non-B hepatitis outbreak in the Kashmir region of the Indian subcontinent [1,2]. There are four major HEV genotypes (HEV G1–4) that can infect humans. Genotypes 1 and 2 only infect humans, are transmitted primarily by a faecal-oral route or contaminated water, and were likely responsible for the first described outbreaks. For more than a decade it has been recognised that autochthonous infections with HEV genotype 3 are common in some industrialised countries [3,4]. Although the transmission routes are not yet completely understood, it is thought that domesticated swine, wild boar and deer are reservoirs for the zoonotic HEV genotype 3 strains and a likely source of human infection through the consumption of uncooked meat, shellfish, vegetables and berries [5-8]. In most cases the infection, especially in immunocompetent individuals, is asymptomatic [9]. The symptomatic clinical signs of an acute HEV infection are nausea, vomiting, malaise, loss of appetite and jaundice (similar clinical presentation as classic hepatitis infection), but protracted neurological signs can also develop, particularly for genotype 3 [10]. Most infections are self-limiting but can, in exceptional cases, develop into a life-threatening fulminant hepatitis, particularly in pregnant women infected with genotype 1 and 2, or in immunocompromised patients infected with genotype 3 [11]. The HEV infection may also develop into a chronic infection in immunocompromised individuals [12]; this has been primarily observed in solid organ recipients, but has also been reported in other immunosuppressed individuals receiving numerous blood components [13].

Transfusion transmission of HEV was previously described with genotype 1 [14] and, more recently, HEV transmission with genotype 3 was reported in several European countries [15,16]. Prevalence of HEV viraemia in donors has been reported to range from 1:762 in the Netherlands to 1:9,500 in the United States [17,18], highlighting the potential for a higher risk of HEV transmission from contaminated blood products than previously assumed, particularly to vulnerable immunosuppressed recipients.

In recent years, a number of published data have indicated a wide variation in HEV seroprevalence in Europe, ranging from 1.9% in Switzerland to 86.4% in France [19,20]. The reason for this wide variation is not entirely clear, but it could be a consequence of many factors, such as eating habits, differences in food production, age of the tested population, country or region of residence or sex. Furthermore, the variable performance of different anti-HEV IgG assays has hindered a direct comparison of the various data.

HEV is currently not a notifiable infectious disease in Switzerland and as a result HEV infections are probably under-diagnosed as they are either not reported or missed due to the common asymptomatic or mild symptomatic presentation. In addition, symptoms compatible with an HEV infection have, until recently, been attributed to a variety of other causes including other viral agents, drug-related or autoimmune hepatitis, ischaemic hepatopathy, hepatic graft-versus-host reaction after stem cell transplantation, or rejection or surgical complication after liver transplantation.

The purpose of this study was to investigate the current and past seroprevalence of anti-HEV IgG in Switzerland and to assess the regional distribution patterns. Furthermore, this study aimed to increase the clinicians’ awareness of HEV infection through contaminated food products, transplantations and blood products, as well as to provide rationale for a possible prospective nationwide HEV donation screening strategy.

## Methods

### Regional distribution of HEV seroprevalence in Swiss blood donors, 2014–2016

**Figure 1:**
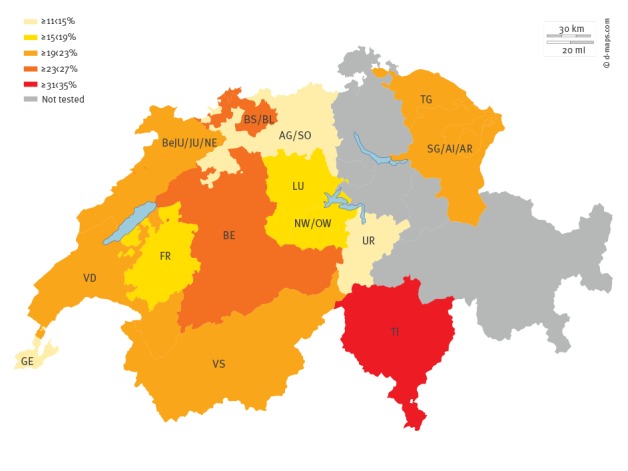
Regional distribution of hepatitis E IgG seroprevalence in blood donors, Switzerland, August 2014–February 2016 (n = 3,609)

Between August 2014 and February 2016, a total of 3,609 EDTA plasma samples were collected from unlinked, unpaid, volunteer blood donors from 20 of the 26 Swiss cantons, including 10 of the 12 regional Blood Transfusion Services (BTS) (Figure 1). Two BTS representing six cantons declined to join the study. However, as all the major geographical regions (e.g. alpine, valley, towns, cities and country borders) encountered in Switzerland are covered by the 20 cantons included in this study, the results are considered generalisable to the whole country. The presence of HEV-specific IgG antibodies were assessed using the anti-HEV IgG assay (WANTAI HEV-IgG ELISA, Eurobio, Les Ulis, France), according to the manufacturer’s instructions, on a BEPIII Behring ELISA processor (Siemens Healthcare Diagnostics, Marburg, Germany). The Wantai HEV IgG EIA has been reported to be the most sensitive and specific [21,22]. Results are expressed as signal-to-cut-off (s/co) value; samples with an s/co value < 0.9 were considered negative and those with an s/co value ≥ 2 were considered positive. Those with s/co values between 0.9 and 2.0 were retested in duplicate and were considered positive if s/co > 1.1, negative if s/co < 0.9 or borderline if s/co was between 0.9 and 1.1 in two of the three replicates, respectively. The age and sex of the donors was obtained from the corresponding regional BTS donor database.

AG/SO: Aargau/Solothurn; BeJU/JU/NE: Berner Jura/Jura/Neuchâtel; BE: Bern; BS/BL: Basel-Stadt/Basel-Landschaft; FR: Fribourg; GE: Genève; LU: Luzern; NW/OW: Nidwalden/Obwalden; SG/AI/AR: St. Gallen/Appenzell Innerrhoden/Appenzell Ausserrhoden; TG: Thurgau; TI: Ticino; UR: Uri; VD: Vaud; VS: Valais.

According to Swissmedic, the federal authority responsible for the notification of blood components, separate donor consent for testing HEV IgG antibodies in blood donors was not required as it is included in the current donor consent form.

### Retrospective study of HEV seroprevalence in blood donors from Bern canton, 1997/1998, 2006, 2015/2016

The anti-HEV IgG seroprevalence (WANTAI HEV-IgG ELISA) among blood donors from Bern canton in 1997/98 (August 1997–November 1998) and 2006 (January–December) was determined on archival samples stored at -30 °C, using the same test criteria as the assessment of regional HEV seroprevalence described previously. From each period, 400 donor samples that were equally distributed within Bern canton, were selected to match the age and sex in 10 age categories with the 400 donor samples collected in 2015/16 (September 2015–June 2016).

### Statistical analysis

The prevalence of anti-HEV IgG antibodies and the 95% confidence intervals (CI) according to Wilson method without continuity correction were calculated. Pearson’s chi-squared test was used to evaluate regional variations and the effect of age on the anti-HEV IgG seroprevalence. The same test was used to assess possible differences between the anti-HEV IgG prevalence rates in 1997/98, 2006 and 2015. Yates’ continuity correction was applied to Pearson’s chi-squared test for pairwise comparisons of 2 years. In order to simultaneously assess the influence of the time (i.e. the year of the test) and the demographic characteristics, these analyses were complemented with multivariate analyses using logistic regression models for anti-HEV IgG prevalence rates during 1997/98, 2006 and 2015. Demographic characteristics incorporated in the multivariate analyses included age, sex and region of residence. A statistical significance was calculated and a p value of less than 0.05 was defined as a statistically significant difference. All calculations were conducted with the software R 3.3.1 (Ref R Core Team. 2016; R Foundation for Statistical Computing, Vienna; http://www.R-project.org).

## Results

In total, there were 3,609 (m = 2,225, w = 1,384) blood samples collected from the 20 cantons in this study, with a mean number of tested blood donor samples of 240 (range: 61–409). The median age of donors was 47 years (interquartile range (IQR): 34–56; range: 17–76).

### Regional distribution of HEV seroprevalence in Swiss blood donors, 2014–2016

The overall positive anti-HEV IgG seroprevalence among the 3,609 Swiss blood donors in 2015/16 was 20.4% (95%CI: 19.1–21.8) (Table1). A statistically significant variation in the HEV IgG seropositivity associated with the different Swiss geographical regions (n = 3,609, 95%CI: 19.1–21.8: p < 0.001) was observed (Table 1 and Figure 1). The lowest levels were observed in the cantons of Geneva (n = 24; 95% CI: 8.7–18.3) and Uri (n = 20; 95% CI: 8.5–19.1), whereas in Ticino canton–the most southern canton situated below the Alps, the highest level was observed (n = 116; 95% CI: 28.8–38.8).

**Table 1:** Regional distribution of hepatitis E IgG prevalence in blood donors, Switzerland, August 2014–February 2016 (n = 3,609)

Region	IgG positive		IgG borderline		IgG negative		Total number of donations
	Number of donations	Prevalence (95% CI)	Number of donations	Prevalence (95% CI)	Number of donations	Prevalence (95% CI)	
GE	24	12.8 (8.7–18.3)	0	0.0 (0.0–2.0)	164	87.2 (81.7–91.3)	188
UR	20	12.9 (8.5–19.1)	2	1.3 (0.4–4.6)	133	85.8 (79.4–90.4)	155
AG/SO	30	13.3 (9.5–18.3)	1	0.4 (0.1–2.5)	195	86.3 (81.2–90.2)	226
NW/OW	58	15.6 (12.3–19.6)	0	0.0 (0.0–1.0)	314	84.4 (80.4–87.7)	372
LU	28	16.0 (11.3–22.2)	0	0.0 (0.0–2.1)	147	84.0 (77.8–88.7)	175
FR	39	18.4 (13.8–24.2)	1	0.5 (0.1–2.6)	172	81.1 (75.3–85.8)	212
BeJU/JU/NE	51	19.5 (15.1–24.7)	1	0.4 (0.1–2.1)	210	80.2 (74.9–84.5)	262
VS	39	19.5 (14.6–25.5)	3	1.5 (0.5–4.3)	158	79.0 (72.8–84.1)	200
TG	39	20.2 (15.1–26.4)	1	0.5 (0.1–2.9)	153	79.3 (73.0–84.4)	193
SG/AI/AR	63	21.5 (17.2–26.6)	3	1.0 (0.3–3.0)	227	77.5 (72.4–81.9)	293
VD	91	22.2 (18.5–26.5)	1	0.2 (0.0–1.4)	317	77.5 (73.2–81.3)	409
BS/BL	56	23.3 (18.4–29.1)	3	1.3 (0.4–3.6)	181	75.4 (69.6–80.4)	240
BE	69	24.8 (20.1–30.2)	1	0.4 (0.1–2.0)	208	74.8 (69.4–79.6)	278
TI	116	33.6 (28.8–38.8)	2	0.6 (0.2–2.1)	227	65.8 (60.6–70.6)	345
Other origin	14	23.0 (14.2–34.9)	0	0.0 (0.0–5.9)	47	77.0 (65.1–85.8)	61
**Total**	**737**	**20.4 (19.1–21.8)**	**19**	**0.5 (0.3–0.8)**	**2,853**	**79.1 (77.7–80.3)**	**3,609**

AG/SO: Aargau/Solothurn; BeJU/JU/NE: Berner Jura/Jura/Neuchâtel; BE: Bern; BS/BL: Basel-Stadt/Basel-Landschaft; CI: confidence interval; FR: Fribourg; GE: Genève; LU: Luzern; NW/OW: Nidwalden/Obwalden; SG/AI/AR: St. Gallen/Appenzell Innerrhoden/Appenzell Ausserrhoden; TG: Thurgau; TI: Ticino; UR: Uri; VD: Vaud; VS: Valais.

The overall performance of the Wantai HEV IgG EIA was determined by the reproducibility of repeated testing of the samples, with s/co values consistently reported between 0.9 and 2.0. Most repeat results (n = 133) corroborated the original result, whereas only 0.25% (n = 10) were re-categorised as negative. The 0.5% (n = 19) borderline samples were excluded from the statistical analysis.

In total, there were 2,217 men and 1,382 women donors, with the most frequent category aged between 51-60 years (male: n = 653; female: n = 319) (Table 2).

**Table 2:** Age distribution of male and female blood donors, Switzerland, August 2014–February 2016 (n = 3,599^a^)

Age group (years)	Male	Female
≤ 30	307	378
31-40	351	202
41-50	580	305
51-60	653	319
≥ 61	326	178
**Total**	**2,217**	**1,382**

^a^The age of eight males and two females are unknown.

**Figure 2:**
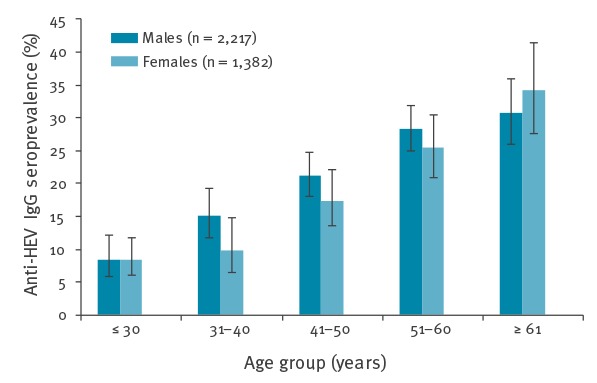
Age and sex distribution of hepatitis E IgG positive blood donors, Switzerland, August 2014–February 2016 (n = 3,599^a^)

^a^The age of eight males and two females are unknown.

A statistically significant steady increase in the HEV seroprevalence of donors (of both sexes) with age was observed, i.e. 8.5% in donors aged < 30 years to 30.7% in donors aged > 60 years (p < 0.001). The non-equivalence in the average age of the male and female donors (male: 47 years, female: 43 years) could in part explain the observed difference between male donors 22.0% (20.3%–23.7%) and female donors 17.9% (15.9%–19.8%) (Figure 2).

Vertical lines indicate 95% confidence intervals.

### Retrospective study of HEV seroprevalence in blood donors from Bern canton, 1997/1998, 2006, 2015/2016

**Figure 3:**
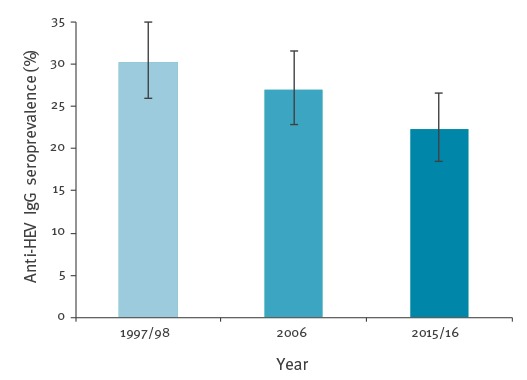
Overall anti-HEV IgG seroprevalence in a matched cohort of blood donor samples from the region of Bern canton, 1997/98, 2006, 2015/16 (n = 400)

**Figure 4:**
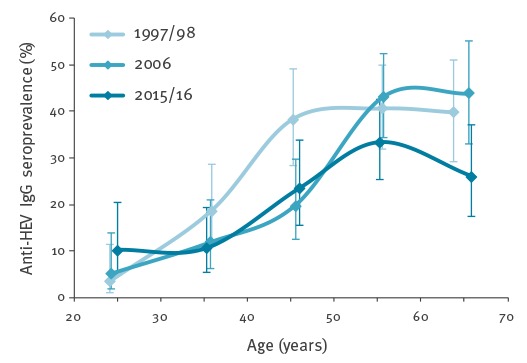
Anti-HEV IgG seroprevalence by age in a matched cohort of blood donor samples from the region of Bern canton, 1997/98, 2006, 2015/16 (n=1,200)

Archival plasma stored at -30 °C were analysed to determine if there was a difference in the HEV seroprevalence from Bern canton during the time periods of 1997/1998 and 2015/2016. The seroprevalence increased with age in all years tested; however, the overall seroprevalence from all years declined significantly from 30.3% (95%CI: 26.0–35.0) in 1997/98 to 27.0% (95%CI: 22.9–31.6) in 2006 and 22.3% (95%CI: 18.5–26.6) in 2015/16 (Pearson chi-squared test over the three time points: p = 0.027) (Figures 3 and 4). The analysis revealed a significant difference between 1997/98 and 2015/16 (p = 0.0093), but between 1997/98 and 2006 (and between 2006 and 2015/16) the difference was not significant (p > 0.05). Analysis of different age groups found a significant reduction in seroprevalence in the 40–50-year-old and > 60-year-old age groups (p = 0.039 and p = 0.038, respectively). Furthermore, since the observed difference between the three time points could have been due to varying demographic characteristics, a logistic regression models were used in order to be able to analyse the simultaneous effect of three parameters (time, age, sex, and/or regional predictors) on the HEV seroprevalence. If only the time as a factor with three levels was used as an influence, the likelihood ratio (LR) test gave a p value = 0.026 for the influence of this factor (LR = 7.2977, df = 2). Assuming a linear trend (more precisely, a linear effect of the time on the logit of the probability of a positive result) over all three time points, a p value of 0.0072 (LR = 7.2232, df = 1) was obtained. When adding main effects of the sex and the age categories to this model, the effect of the time was still significant (LR = 8.366, df = 1, p = 0.0038). No significant interaction effects between the time, the sex, and the age groups were found. In extensions of the model with these three main effects (time, sex, and age category) that additionally included one of several possible predictors based on the place of residence, the p value of the LR test for the effect of the time was always between 0.001 and 0.008. Thus, a significant reduction in the HEV IgG seroprevalence over time was found in all the models used.

Pearson’s chi-squared test: p = 0.027 between all the time points; p = 0.009 between 1997/98 and 2015/16; p = 0.324 between 1997/98 and 2006; p = 0.124 between 2006 and 2015/16.

Vertical lines indicate 95% confidence intervals.

Vertical lines indicate 95% confidence intervals.

Age: averaged over 10-year age categories.

## Discussion

Anti-HEV IgG seroprevalence data from various countries indicate that prior HEV infection has been increasing during the last decade. This increase may reflect a real rise in HEV incidence, particularly in European countries, but could also be a reaction to an increased public health awareness of HEV infection acquired through zoonotic and food-borne transmission of HEV. The situation has been further complicated by the broad performance variation of the currently available commercial anti-HEV assays [23]. The Wantai HEV IgG EIA is regarded as one of the most sensitive and specific assays currently commercially available [21,22].

There have been a limited number of HEV seroprevalence studies on blood donations in Switzerland, but these have generally been restricted to a confined region, have tested a limited number of samples and have used less sensitive assays [24-26]. These reports revealed a wide difference in the seroprevalence (4.2%–21.8%), with no difference between male and female donors. This present study has revealed an overall seroprevalence of 20.4% (95%CI: 19.1–21.8), with significant differences between the regions tested. As expected from other published reports, the anti-HEV seroprevalence increased significantly with age. The overall seroprevalence difference observed between the sex of the donors is likely a consequence of the differing average age of these donors, however this observation was not supported in the multivariate analysis using time/age/sex simultaneously.

In many European countries similarly high seroprevalence differences have been recorded; for example, 25%–86.4% in France and 21%–27% in the Netherlands [19,20,27,28]. Interestingly, the French Jura, a region directly adjacent to the west of Switzerland, has reported a similar seroprevalence to the one in our report [20]. High prevalence has not been observed in all industrialised countries, for example in Scotland, Canada and Australia prevalence did not exceed 6% [29,30], and within countries a considerable variation has often been observed. Variations in HEV IgG seroprevalence were also observed in our study (range: 12.8%–33.6%) and we found that in some isolated districts within the Ticino canton it was bordering on 60% (data not shown). It has been suggested that these large regional variations may be a consequence of the consumption of a regional delicacy of traditional raw dry-cured pork sausage containing raw pork liver (e.g. *mortadella di fegato crudo*) [19,31]. It remains to be seen whether the variation in Switzerland can be explained by these dietary differences.

In some countries, notably Denmark, the Netherlands and the United States, the analysis of archived samples has suggested the seroprevalence has declined in recent decades [32-35], whereas other countries have reported an increase [36]. The analysis of 400 archived blood donor samples from Bern canton from 1997/98, 2006 and 2015/16 showed a similar declining anti-HEV IgG prevalence from 30.3% in 1997/98 to 27.0% in 2006 and 22.3% in 2015/16. The trends in seroprevalence in other studies has been questioned due to the differences in the sensitivities and specificities of the tests used [32,34]. Since the results presented here were all conducted with the same EIA, and often in the same experiment, the observed seroprevalence reduction over time appears to be real and may reflect a change in the level of contaminated food, which is regarded as the primary route of acquiring HEV infection. It remains to be seen whether the slight anti-HEV IgG increase in the most recent samples from the < 30-years-old age group reflects an increase in the HEV infection of these individuals.

HEV seroprevalence studies are important to highlight the level of infections within a population and to contribute to assessments of the epidemiological situation within the population. Likewise, HEV RNA incidence studies of blood donors contribute to the risk determination of HEV transmission via blood products. In Europe, incidences ranging from 1:600 to 1:15,000 have been recently reported [16,17,37], and several studies have documented HEV blood transfusion transmission [15,16,38,39]. Though many HEV transfusion-transmitted infections are asymptomatic and self-limiting, particularly in immunocompetent patients, serious chronic infections can occur in immunosuppressed patients (e.g. solid organ and haematopoietic stem cell transplant patients). It is often these patients who receive numerous blood products and are thus at greater risk to HEV infection. Several European countries (e.g. France, Ireland, the Netherland and the United Kingdom) [40] have already begun molecular screening of blood donations for HEV RNA in different formats (individual donation or minipools). In Switzerland, the seroprevalence data presented here have recently contributed to discussions within the Swiss blood transfusion community; together with the national health authority and clinical hepatologists, they have come up with recommendations for future HEV molecular testing of blood donations and the consequent monitoring of at-risks patients. As a consequence of these discussions, from November 2018 all blood products will be mandatorily screened in Switzerland for HEV RNA in pools of 24 or less blood donations. The required detection limit in the individual donation was set at 450 HEV IU/ml.

## References

[1] Khuroo MS Chronic liver disease after non-A, non-B hepatitis. Lancet. 1980;2(8199):860-1. . Available from 10.1016/S0140-6736(80)90205-6 6107528

[2] Khuroo MS Discovery of hepatitis E: the epidemic non-A, non-B hepatitis 30 years down the memory lane. Virus Res. 2011;161(1):3-14. 10.1016/j.virusres.2011.02.007 21320558

[3] Schlauder GG Desai SM Zanetti AR Tassopoulos NC Mushahwar IK Novel hepatitis E virus (HEV) isolates from Europe: evidence for additional genotypes of HEV. J Med Virol. 1999;57(3):243-51. 10.1002/(SICI)1096-9071(199903)57:3<243::AID-JMV6>3.0.CO;2-R 10022795

[4] Clemente-Casares P Pina S Buti M Jardi R MartIn M Bofill-Mas S Hepatitis E virus epidemiology in industrialized countries. Emerg Infect Dis. 2003;9(4):448-54. 10.3201/eid0904.020351 12702225 PMC2957966

[5] Kamar N Bendall R Legrand-Abravanel F Xia NS Ijaz S Izopet J Hepatitis E. Lancet. 2012;379(9835):2477-88. 10.1016/S0140-6736(11)61849-7 22549046

[6] Meng XJ Zoonotic and foodborne transmission of hepatitis E virus. Semin Liver Dis. 2013;33(1):41-9. 10.1055/s-0033-1338113 23564388

[7] Crossan C Baker PJ Craft J Takeuchi Y Dalton HR Scobie L Hepatitis E virus genotype 3 in shellfish, United Kingdom. Emerg Infect Dis. 2012;18(12):2085-7. 10.3201/eid1812.120924 23171845 PMC3557861

[8] Kokkinos P Kozyra I Lazic S Bouwknegt M Rutjes S Willems K Harmonised investigation of the occurrence of human enteric viruses in the leafy green vegetable supply chain in three European countries. Food Environ Virol. 2012;4(4):179-91. 10.1007/s12560-012-9087-8 23412890

[9] Dalton HR Kamar N Izopet J Hepatitis E in developed countries: current status and future perspectives. Future Microbiol. 2014;9(12):1361-72. 10.2217/fmb.14.89 25517900

[10] Dalton HR Hunter JG Bendall RP Hepatitis E. Curr Opin Infect Dis. 2013;26(5):471-8. 23982238 10.1097/01.qco.0000433308.83029.97

[11] Salam GD Kumar A Kar P Aggarwal S Husain A Sharma S Serum tumor necrosis factor-alpha level in hepatitis E virus-related acute viral hepatitis and fulminant hepatic failure in pregnant women. Hepatol Res. 2013;43(8):826-35. 10.1111/hepr.12028 23279190

[12] Kamar N Selves J Mansuy JM Ouezzani L Péron JM Guitard J Hepatitis E virus and chronic hepatitis in organ-transplant recipients. N Engl J Med. 2008;358(8):811-7. 10.1056/NEJMoa0706992 18287603

[13] Riezebos-Brilman A Puchhammer-Stöckl E van der Weide HY Haagsma EB Jaksch P Bejvl I Chronic hepatitis E infection in lung transplant recipients. J Heart Lung Transplant. 2013;32(3):341-6. 10.1016/j.healun.2012.11.027 23415316

[14] Khuroo MS Kamili S Yattoo GN Hepatitis E virus infection may be transmitted through blood transfusions in an endemic area. J Gastroenterol Hepatol. 2004;19(7):778-84. 10.1111/j.1440-1746.2004.03437.x 15209625

[15] Colson P Coze C Gallian P Henry M De Micco P Tamalet C Transfusion-associated hepatitis E, France. Emerg Infect Dis. 2007;13(4):648-9. 10.3201/eid1304.061387 17561564 PMC2725983

[16] Hewitt PE Ijaz S Brailsford SR Brett R Dicks S Haywood B Hepatitis E virus in blood components: a prevalence and transmission study in southeast England. Lancet. 2014;384(9956):1766-73. 10.1016/S0140-6736(14)61034-5 25078306

[17] Hogema BM Molier M Sjerps M de Waal M van Swieten P van de Laar T Incidence and duration of hepatitis E virus infection in Dutch blood donors. Transfusion. 2016;56(3):722-8. 10.1111/trf.13402 26559806

[18] Stramer SL Moritz ED Foster GA Ong E Linnen JM Hogema BM Hepatitis E virus: seroprevalence and frequency of viral RNA detection among US blood donors. Transfusion. 2016;56(2):481-8. 10.1111/trf.13355 26434952

[19] Ankcorn MJ Tedder RS Hepatitis E: the current state of play. Transfus Med. 2017;27(2):84-95. 10.1111/tme.12405 28382704

[20] Mansuy JM Gallian P Dimeglio C Saune K Arnaud C Pelletier B A nationwide survey of hepatitis E viral infection in French blood donors. Hepatology. 2016;63(4):1145-54. 10.1002/hep.28436 27008201

[21] Bendall R Ellis V Ijaz S Ali R Dalton H A comparison of two commercially available anti-HEV IgG kits and a re-evaluation of anti-HEV IgG seroprevalence data in developed countries. J Med Virol. 2010;82(5):799-805. 10.1002/jmv.21656 20336757

[22] Wenzel JJ Preiss J Schemmerer M Huber B Jilg W Test performance characteristics of Anti-HEV IgG assays strongly influence hepatitis E seroprevalence estimates. J Infect Dis. 2013;207(3):497-500. 10.1093/infdis/jis688 23148290

[23] Hartl J Otto B Madden RG Webb G Woolson KL Kriston L Hepatitis E Seroprevalence in Europe: A Meta-Analysis. Viruses. 2016;8(8):211. 10.3390/v8080211 27509518 PMC4997573

[24] Schnegg A Bürgisser P André C Kenfak-Foguena A Canellini G Moradpour D An analysis of the benefit of using HEV genotype 3 antigens in detecting anti-HEV IgG in a European population. PLoS One. 2013;8(5):e62980. 10.1371/journal.pone.0062980 23667554 PMC3646942

[25] Kaufmann A Kenfak-Foguena A André C Canellini G Bürgisser P Moradpour D Hepatitis E virus seroprevalence among blood donors in southwest Switzerland. PLoS One. 2011;6(6):e21150. 10.1371/journal.pone.0021150 21701586 PMC3118806

[26] Gottschalk J, Hardegger K, Darnuzer R, Frey B. Seroprevalence of Hepatitis E virus in Swiss blood donors originating from the canton of Zürich. SGM-Jahrestagung; Interlaken 2013. Available from http://www.blutspendezurich.ch/Media/File/Archiv%20div.%20Daten/HEV%20SGM%202013%20Kompatibilit%C3%A4tsmodus.pdf

[27] Mansuy JM Bendall R Legrand-Abravanel F Sauné K Miédouge M Ellis V Hepatitis E virus antibodies in blood donors, France. Emerg Infect Dis. 2011;17(12):2309-12. 10.3201/eid1712.110371 22172156 PMC3311200

[28] Slot E Hogema BM Riezebos-Brilman A Kok TM Molier M Zaaijer HL Silent hepatitis E virus infection in Dutch blood donors, 2011 to 2012. Euro Surveill. 2013;18(31):20550. 10.2807/1560-7917.ES2013.18.31.20550 23929229

[29] Cleland A Smith L Crossan C Blatchford O Dalton HR Scobie L Hepatitis E virus in Scottish blood donors. Vox Sang. 2013;105(4):283-9. 10.1111/vox.12056 23763589

[30] Fearon MA O’Brien SF Delage G Scalia V Bernier F Bigham M Hepatitis E in Canadian blood donors. Transfusion. 2017;57(6):1420-5. 10.1111/trf.14089 28394029

[31] Kubacki J Fraefel C Jermini M Giannini P Martinetti G Ripellino P Complete Genome Sequences of Two Swiss Hepatitis E Virus Isolates from Human Stool and Raw Pork Sausage. Genome Announc. 2017;5(35):e00888-17. 10.1128/genomeA.00888-17 28860248 PMC5578846

[32] Holm DK Moessner BK Engle RE Zaaijer HL Georgsen J Purcell RH Declining prevalence of hepatitis E antibodies among Danish blood donors. Transfusion. 2015;55(7):1662-7. 10.1111/trf.13028 25819381

[33] Ijaz S Vyse AJ Morgan D Pebody RG Tedder RS Brown D Indigenous hepatitis E virus infection in England: more common than it seems. J Clin Virol. 2009;44(4):272-6. 10.1016/j.jcv.2009.01.005 19217345

[34] Hogema BM Molier M Slot E Zaaijer HL Past and present of hepatitis E in the Netherlands. Transfusion. 2014;54(12):3092-6. 10.1111/trf.12733 24889277 PMC4280434

[35] Wenzel JJ Sichler M Schemmerer M Behrens G Leitzmann MF Jilg W Decline in hepatitis E virus antibody prevalence in southeastern Germany, 1996-2011. Hepatology. 2014;60(4):1180-6. 10.1002/hep.27244 24912687

[36] Faber MS Wenzel JJ Jilg W Thamm M Höhle M Stark K Hepatitis E virus seroprevalence among adults, Germany. Emerg Infect Dis. 2012;18(10):1654-7. 10.3201/eid1810.111756 23018055 PMC3471611

[37] Baylis SA Koc O Nick S Blümel J Widespread distribution of hepatitis E virus in plasma fractionation pools. Vox Sang. 2012;102(2):182-3. 10.1111/j.1423-0410.2011.01527.x 21806631

[38] Huzly D Umhau M Bettinger D Cathomen T Emmerich F Hasselblatt P Transfusion-transmitted hepatitis E in Germany, 2013. Euro Surveill. 2014;19(21):20812. 10.2807/1560-7917.ES2014.19.21.20812 24906377

[39] Djoudi RD, Gallian PG, Roque-Afonso AMRA, Bierling PB, Assal AA, Hauser LH, et al. Occurrence of Transfusion-Transmitted Hepatitis E in France. Vox Sang 2015;109 (Suppl. 1)(4C-S22-02):1-379.

[40] Domanović D Tedder R Blümel J Zaaijer H Gallian P Niederhauser C Hepatitis E and blood donation safety in selected European countries: a shift to screening? Euro Surveill. 2017;22(16):30514. 10.2807/1560-7917.ES.2017.22.16.30514 28449730 PMC5404480

